# Antimicrobial activity of carbon dots against aquatic spoilage Bacteria synthesized from Banana Peel waste

**DOI:** 10.1016/j.fochx.2025.102375

**Published:** 2025-03-15

**Authors:** Yueyue Meng, Huiyu Zhu, Xinyi Li, Shuang Zhao, Kun Ma, Tingting Li

**Affiliations:** Key Laboratory of Biotechnology and Bioresources Utilization (Dalian Minzu University), Ministry of Education, Dalian, Liaoning 116600, China

**Keywords:** Banana peels, Carbon dots, Spoilage bacteria, Metabolomics

## Abstract

In this study, we used biomass banana peel (BP) as the precursor to synthesize CDs (defined as BP-CDs). The average sizes of BP-CDs were found to be around 5.9 nm. The prepared BP-CDs presented blue fluorescence under ultraviolet irradiation at 365 nm and exhibited excitation-wavelength-dependent fluorescence properties. Furthermore, the nitrogen-containing, oxygen-containing and sulfur-containing functionalities on/in the surface of carbon structure were observed in the resulting CDs. The antioxidant assays in vitro suggested that BP-CDs displayed strong free-radical scavenging abilities. The results of antimicrobial experiments revealed that BP-CDs exhibited noteworthy antimicrobial effects against spoilage bacteria of aquatic products. Metabolomics analysis revealed that BP-CDs exerted antibacterial activity mainly by impeding normal cell metabolism, disrupting cell wall integrity, affecting substance transport and signal transduction. Taken together, these data suggest that BP-CDs could be used as an antioxidant and antibacterial agent for the development of new preservation technologies for aquatic products.

## Highlights

• Banana peels-derived carbon dots (BP-CDs) were synthesized.
• BP-CDs exhibited noteworthy radical scavenging activity.
• BP-CDs had antibacterial effect against the spoilage bacteria of aquatic products.
• Metabolomics analysis was performed to reveal the antibacterial mechanism of BP-CDs.

## 1 Introduction

Aquatic products, typically harvested at a relatively concentrated time, are prone to oxidation and microbial contamination during storage and transportation. This issue compromises the flavor of aquatic products and significantly threatens food safety and human health (Roy, Park, Song, & Park, 2022). Aquatic products have high amounts of protein, while being low in carbohydrates, which make microorganisms responsible for aquatic products spoilage different from other food. It has been reported that Gram-negative psychrotrophic bacteria, including *Pseudomonas*, *Aeromonas*, and *Shewanella*, etc. are major contributors to the spoilage of aquatic products, which were identified as specific spoilage organisms (SSOs) (Liu et al., 2018). Accordingly, inhibiting the growth and reproduction of SSOs can be crucial for preserving aquatic products. To delay the deterioration of aquatic products, chemically synthesized fresh-keeping agents like chlorine dioxide has been widely utilized as a preservative. However, the usage of chemical compounds to protect food products during preservation is becoming increasingly limited with growing concerns about public health and food safety (Olatunde & Benjakul, 2018). Thus, developing novel alternative additive agents with antioxidant and antimicrobial abilities that are safe and effective is crucial for preserving aquatic products.

Carbon dots (CDs) are a kind of emerging nanomaterials with a diameter smaller than 10 nm. Owing to their fascinating characteristics, such as extremely small size, good aqueous solubility, outstanding biocompatibility and ease of synthesis (Cui et al., 2024), CDs have earned tremendous attention and been applied to various fields e.g., biological imaging (Zhang et al., 2019), drug carrying (Kailasa, Bhamore, Koduru, & Park, 2019), sensing (Hu, Gao, & Luo, 2021) and catalysis (Chen, Liu, & Huang, 2020). The aforementioned CDs are typically synthesized with chemical substances including organic solvents, inorganic salts, amines, and organic acids. Despite the promising antimicrobial efficacy of CDs synthesized using chemical reagents, safety concerns of raw materials as well as high costs have hindered their application. Natural biomass and biowaste, derived from plant or animal byproducts, offer significant advantages over chemical precursors for CD synthesis. These benefits include low cost, practicality, environmental friendliness, high biocompatibility, and reproducibility. For example, the CDs prepared from *Artemisia argyi* leaves has been reported to possess antibacterial activity against Gram-negative bacteria (Wang et al., 2020). Panda et al. designed an antibacterial gel based on papaya leaf CDs with antimicrobial effect against both Gram-positive and Gram-negative bacteria (Panda, ChawPattnayak, Dash, Nayak, & Mohapatra, 2021). Banana peel (BP), one of the by-products of bananas, constitutes 30 % to 40 % of the total weight of the banana. The presence of various bioactive compounds such as dietary fiber and phenolic chemicals in banana peels suggests that the peels have multiple bioactivities including antioxidant and antibacterial properties (Zou, Tan, Zhang, Wu, & Shang, 2022). However, most of these peels are often disposed of in landfills or next to other waste, leaving a large amount of this valuable raw material unused.

In the present study, BP was adopted as a precursor to prepare CDs through a facile hydrothermal method. The structures and features of the obtained CDs (defined as BP-CDs) were characterized via transmission electron microscopy (TEM), X-ray diffraction (XRD), Raman spectroscopy, ultraviolet-visible (UV–vis), fluorescence spectroscopy, Fourier transform infrared spectroscopy (FT-IR) and X-ray photoelectron spectroscopy (XPS). The BP-CDs's biological activities, including antioxidant potential and the antimicrobial activity against spoilage bacteria of aquatic products were determined. Furthermore, mechanisms of antibacterial action of BP-CDs were explored by metabolomics approaches. The results of this work provide a theoretical basis for the further application of CDs in the field of fresh-keeping technologies of aquatic products.

## 2 Material and methods

### 2.1 Materials

Banana peels were provided by the campus supermarket. *Aeromonas sobria* (*A. sobria*), *Hafnia alvei* (*H. alvei*) and *Pseudomonas fluorescens* (*P. fluorescens*) strains were generously gifted by Dr. Fangchao Cui (Bohai University, China). LB broth and agar media were obtained from Qingdao Hope Biol Co., Ltd. All other chemicals were used of high purity grade.

### 2.2 Synthesis of BP-CDs

The CDs were prepared through a hydrothermal method utilizing banana peel waste as the precursor source. In brief, banana peels (15.0 g) were washed, chopped into pieces, and then mashed with a mortar and pestle and transferred to a 50 mL beaker. Subsequently, deionized water (30 mL) was added and homogenized for 3 min at a high speed of 10,000 r/min, and the obtained homogenate was transferred into a Teflon-lined autoclave (50 mL). Then, the autoclave was kept at 200 °C for 8 h in an electron oven, then cooled to ambient temperature. Subsequently, the obtained product was filtered through a filter membrane (0.22 μm), and the resulting solution was dialyzed for 48 h in dialysis bags (500 Da molecular weight cutoff) to remove unreacted precursors. Subsequently, the solution in the dialysis bag was freeze-dried using a lyophilizer (FD-1 A-50+; Biocool, Ltd., Beijing, China) to obtain BP-CDs powder.

### 2.3 Characterization of BP-CDs

#### 2.3.1 TEM analysis

The ultrastructure of BP-CDs was examined by TEM. Briefly, 10 μL of BP-CDs solution (2 mg/mL) was dripped onto copper grids. After drying the copper mesh with infrared lamp, the sample was visualized with a JEOL JEM-1011 TEM (Tokyo, Japan) at 60 kV.

#### 2.3.2 Particle size analysis

The concentration of BP-CDs solution was adjusted using deionized water to a certain concentration. Then, the particle size of BP-CDs was detected using a particle size analyzer (SZ-100, Kyoto, Japan).

#### 2.3.3 XRD analysis

The BP-CDs powder was evenly spread in a sample pan and analyzed using a Shimadzu XRD-6000 X-ray diffractometer (Kyoto, Japan).

#### 2.3.4 Raman spectra analysis

BP-CDs solution of 50 μL was dropped onto a slide and the Raman spectrum was collected by a confocal micro Raman spectrometer (Renishaw, *InVia*, UK).

#### 2.3.5 UV–vis spectra analysis

The BP-CDs solution was appropriately diluted with the help of deionized water to make an absorbance of 1.0 at 280 nm, and monitored using a UV–Vis spectrometer (UV-6100, Shanghai Meipuda Instrument Co., Ltd., Shanghai, China).

#### 2.3.6 Fluorescence spectra analysis

The BP-CDs solution was diluted 20 times with deionized water. The fluorescence emission spectra were acquired under different excitation wavelengths using a spectrofluorimeter (Hitachi Co., Ltd., Japan).

#### 2.3.7 FTIR analysis

1 mg of BP-CDs was blended with KBr powder at a mass ratio of 1: 100 and then ground evenly and pressed into a thin slice for detection by an FTIR spectrometer (IR Affinity-1, Shimadzu, Kyoto, Japan).

#### 2.3.8 XPS analysis

The surface elemental composition of BP-CDs was obtained by a K-Alpha XPS spectrometer. (Thermo Fisher Scientific, USA).

### 2.4 Antioxidant activity evaluation

#### 2.4.1 DPPH assay

The test of the DPPH radical scavenging ability was carried out as previously reported (Sachdev & Gopinath, 2015). 1 mL of BP-CDs solution at varying concentrations were mixed with 1 mL of ethanol solution containing 0.1 mM DPPH. The absorbance was determined at 517 nm after 20 min of incubation using a Synergy H1 microplate reader (BioTek).

The DPPH radical scavenging activity of BP-CDs was computed following the equation: DPPH radical scavenging activity (%) = (1 − A_i_/A_0_) × 100, where A_i_ and A_0_ are the absorbance with and without the addition of BP-CDs.

#### 2.4.2 ABTS assay

The ABTS assay of BP-CDs was conducted following the method of Spagnol et al. with some modifications (Spagnol et al., 2019). The ABTS stock solution was prepared as previously described. 50 μL of BP-CDs solution at various concentrations was added to 150 μL of the diluted ABTS solution mixed well, and incubated for 10 min. Then the absorbance of the mixture was read at 734 nm. The ABTS radical scavenging ability of BP-CDs was calculated using a formula as follows: ABTS radical scavenging activity (%) = (1 − A_i_/A_0_) × 100, where A_i_ and A_0_ are the absorbance with and without the addition of BP-CDs.

#### 2.4.3 Hydroxyl assay

The hydroxyl radical (·OH) scavenging activity of BP-CDs was performed by employing the Fenton reaction (Du et al., 2020). In brief, 100 μL of 1.8 mM salicylic acid ethanol solution was mixed well with 200 μL of 7.5 mM ferrous sulfate solution, and 100 μL of BP-CDs at various concentrations were added successively. Then, 900 μL of deionized water was added and stirred followed by addition of 200 μL of 200 mM H_2_O_2_ solution. The absorbance of the mixture was measured at 510 nm after 30 min incubation at 37 °C. The ·OH scavenging capacity of BP-CDs was calculated as follows: hydroxyl radical scavenging capacity (%) = (1 − A_i_/A_0_) × 100, where A_i_ and A_0_ are the absorbance with and without the addition of BP-CDs.

#### 2.4.4 Superoxide anion assay

The activity of BP-CDs to scavenge superoxide anion radical (O_2_^•−^) was measured on the basis of a previously described method (Bae & Suh, 2007). 500 μL of 0.4 mM xanthine was added to 500 μL of 0.24 mM nitro-blue tetrazolium (NBT) and mixed well. Then 1 mL of xanthine oxidase solution (0.049 U/mL) was added, followed by adding 500 μL of BP-CDs at various concentrations. The absorbance of the mixture was measured at 550 nm following a 30-min incubation at 37 °C. The O_2_^•−^ scavenging capacity of BP-CDs was calculated according to the following formula: superoxide anion radical (O_2_^•−^) scavenging capacity (%) = (1 − A_i_/A_0_) × 100, where A_i_ and A_0_ are the absorbance with and without the addition of BP-CDs.

### 2.5 Antibacterial activity evaluation

#### 2.5.1 Bacterial culture

*A. sobria*, *H. alvei* and *P. fluorescens* strains were obtained from Bohai University. The trains were stored at −20 °C and cultured in LB broth medium before use in antibacterial experiments.

#### 2.5.2 Inhibition zone method

The antimicrobial activities of BP-CDs against the strains listed above were tested using the disc diffusion assay. BP-CDs was dissolved in sterilized water and prepared into a 2 mg/mL solution. 100 μL of bacterial suspension was spread onto an LB agar plate. Then sterilized paper disks were placed on the inoculated plates and 50 μL of the prepared BP-CDs was added onto each paper disk. The inoculated plates were subsequently placed in an incubator set at 37 °C. After being cultured for 24 h, the diameters of bacteriostatic rings were measured. Results are expressed as mean ± SD. The antimicrobial activity of BP-CDs was expressed on diameter of inhibition zones (mm).

#### 2.5.3 Spread plate method

The antibacterial activities of BP-CDs against the three strains were further evaluated using the spread plate method. In brief, the bacterial cells (1 × 10^8^ CFU/mL) were prepared and incubated with BP-CDs of a series of concentrations at 37 °C for 12 h. Thereafter, 40 μL of 1000-fold diluted bacterial suspensions spread evenly onto the LB agar media and cultured at 37 °C for 24 h. Control plates were seeded with bacteria that were not treated with BP-CDs.

#### 2.5.4 Live/dead bacteria cell staining

In order to determine the plasma membrane permeability of the tested strains, the live/dead cell staining assay was conducted using SYTO9 (green) and PI (red). First, the bacterial cells (10^7^–10^8^ CFU/mL) were treated with different concentrations of BP-CDs for 2 h, and LB broth medium was then removed by centrifugation at 5000 rpm for 3 min. Next, the bacteria were dyed with SYTO9 and PI for 30 min and washed three times with PBS. Last, images of the stained bacterial suspensions were captured using a Nikon A1R confocal microscope.

#### 2.5.5 SEM characterization of bacteria

In order to probe the effect of BP-CDs on bacterial cells, the morphological alterations of three tested strains with and without the addition of BP-CDs were detected using SEM (Sun et al., 2019). The tested bacterial strains were treated with BP-CDs at concentrations of 0.3 and 0.6 mg/mL. The control groups contained the incubated bacteria without BP-CDs treatment. After incubation at 37 °C for 6 h, the bacterial cells were harvested by centrifugation (6000 rpm, 10 min). Then the samples of bacterial cells were prepared on a silicon chips, fixed with glutaraldehyde 2.5 % (*v*/v) in phosphate buffer. The bacterial cells were sequentially dehydrated with gradient ethanol (30 %, 50 %, 70 %, 90 %, and 100 %), and then sputter-coated with gold after being dried under vacuum. Finally, the morphologies of the as-prepared samples were characterized by using SEM (Nano SEM-450, FEI, America).

#### 2.5.6 Determination of cell membrane permeability

Changes in relative conductivity of bacteria solution before and after treatment with BP-CDs was used to reflect the change of bacterial membrane permeability. The bacterial suspension was centrifuged for 10 min at a rotating speed of 8000 ×g, and the bacterial precipitates were collected, washed and re-suspended with 5 % glucose solution, and conductivity was immediately determined and recorded as L_1_. It was then recorded as L_2_ after incubating three bacteria solutions with different concentrations of BP-CDs for 2 h. Finally, the samples were heated in boiling water for 10 min, and conductivity was measured and recorded as L_0_. Relative conductivity calculation was performed according to the formula below:

Relative conductivity (%) = (L_2_−L_1_)/L_0_ × 100.

#### 2.5.7 Determination of released UV-absorbing material of bacteria

Cell membrane damage of three bacteria were evaluated by determining the leakage of intracellular material absorbing at 260 and 280 nm after the treatment with BP-CDs. The bacterial cells (10^7^–10^8^ CFU/mL) were prepared and incubated with BP-CDs at room temperature for 2 h. Afterwards, the samples were centrifuged at 8000 ×*g* for 10 min, and the OD at 260 and 280 nm of the supernatant was measured, which were interpreted to be the leakages of nucleic acid and protein from the bacteria, respectively.

### 2.6 Cellular toxicity test

3 T3-L1 preadipocytes (10^4^cells per 200 μL) incubated with BP-CDs (0, 0.6, 1.2, 1.8, 2.4, and 3 mg/mL) for 1 day. Thereafter, cells were washed with phosphate-buffered saline (PBS) three times. Then, cells were reacted with 20 μL of CCK-8 solution for 4 h. Absorbance was read at 450 nm.

### 2.7 Metabolomics analysis

Untargeted metabolomic profiling service was provided by Biomarker Technologies Co，LTD (Beijing, China). Briefly, the supernatants were collected from *P. fluorescens* and *P. fluorescens* after exposure to BP-CDs and subsequently precipitated in methanol for analysis. Metabolite analysis was performed using Rapid Separation Liquid Chromatography (Thermo Fisher Scientific, USA) and Q Exactive (Thermo, USA). The data were analyzed using Progenesis QI software (Nonlinear Dynamics, Newcastle, United Kingdom). The metabolites were identified in the KEGG, HMDB and Lipid Maps databases as the threshold of VIP > 1, *p*-value <0.05 and |log_2_FC| > 0. Pathway enrichment analysis of metabolites was achieved by the Metaboanalyst 5.0.

### 2.8 Statistical analysis

A triplicate of each assay was performed for three independent studies, and the results are presented as the means ± SD (standard deviation). Statistical analysis was performed by one-way analysis of variance (one-way ANOVA) using SPSS 18.0 software.

## 3 Results and discussion

### 3.1 Characterization of BP-CDs

In this study, the CDs were prepared from banana peels and its morphology and structure were investigated using a series of characterization.

**Fig. 1:**
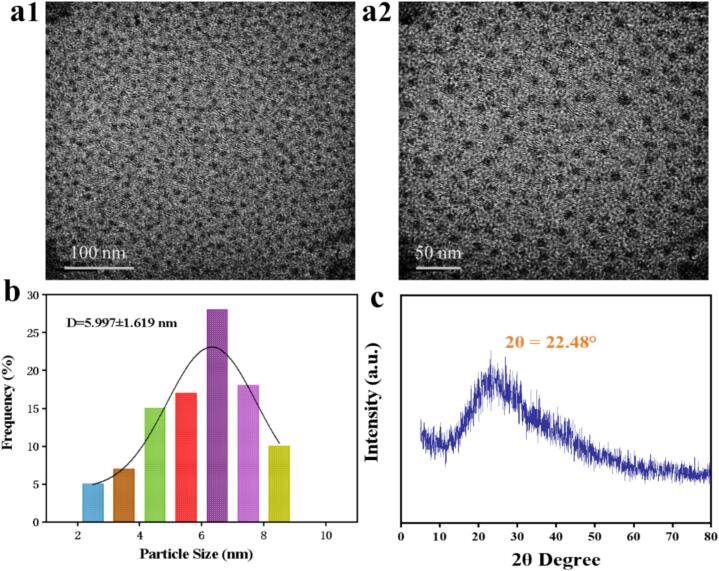
(a) The TEM images, (b) size distribution histogram, and the XRD spectrum of BP-CDs.

**Fig. 2:**
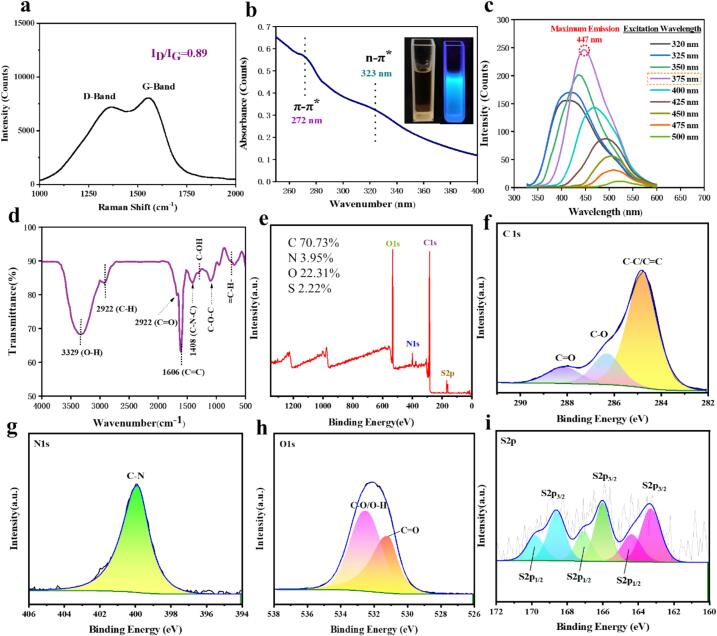
(a) Raman spectrum, (b) UV–vis spectrum, (c) fluorescence emission spectra, (d) FTIR spectrum, (e) XPS survey spectrum (f) high-resolution C1s spectrum, (g) high-resolution N1s spectrum, (h) high-resolution O1s spectrum and (i) high-resolution S2_P_ of BP-CDs.

Fig. 1a shows the TEM image of the synthesized CDs, which clearly showed that the BP-CDs had spherical structures, uniform size distributions and almost identical lattice fringes. The result for particle size distribution is presented in Fig. 1b. The size of BP-CDs ranged from 2.5 to 8.5 nm with the average value of 5.9 nm. The XRD pattern of BP-CDs exhibits a broad diffraction peak at 2θ = 22.48° as shown in Fig. 1c, corresponding to the (002) planes of carbon-based materials (Atchudan, Edison, Sethuraman, & Lee, 2016). Furthermore, Raman spectroscopy was used to further characterize the graphitization degree of BP-CDs. As illustrated in Fig. 2a, the Raman spectrum revealed two broad peaks at 1362 cm^−1^ and 1555 cm^−1^, which are assigned to the graphite peak and disorder-related peak, respectively. The peak at 1362 cm^−1^ is called the D-band, which is attributed to the defects induced in the sp2 carbon nanostructure; and the G band (at 1555 cm^−1^) represents the tangential vibrations the sp2-hybridized carbon (Seo et al., 2017). The intensity ratio of D-band to G-band (ID/IG) is often used to reflect the crystalline quality of carbon materials, and the smaller the ID/IG value, the better the structural quality (Tang et al., 2022). As calculated, the ID/IG ratio of BP-CDs was 0.89, which implied that the graphitic shells have a sufficiently high integrity to protect the core material, to some extent, from corrosion and oxidation.

The electron transition properties of BP-CDs were determined by the UV − vis spectrum. As presented in Fig. 2b, the absorption peak near 272 nm was assigned to the π–π* electronic transition of aromatic C = C bond, and a broad absorption band centered at around 323 nm was ascribed to the n–π* transition of C = O bond (Sun et al., 2020). The inset shows that BP-CDs had a yellowish color in daylight, while showed bright blue fluorescence under UV light irradiation (λ=365 nm).

Fig. 2c shows the fluorescence emission spectra of BP-CDs aqueous solution at excitation wavelengths ranging from 320 to 500 nm. It was observed that the fluorescence intensities of BP-CDs incrementally increased with increasing excitation wavelength from 320 nm to 375 nm, the maximum fluorescence emission intensity being observed at the excitation wavelength of 375 nm, and further raising the excitation wavelength to 500 nm leads to a reduction in emission intensity, exhibiting an excitation-dependent emission behavior. In addition, the emission maxima showed red-shift (long-wavelength shift) with increasing excitation wavelength from 320 nm to 500 nm. These results are in agreement with the emission spectroscopy of the CDs according to the literature (Fu et al., 2015).

The FTIR spectrum presented in Fig. 2d expresses the surface functional groups of BP-CDs. The broad and strong band observed around 3400–3240 cm^−1^ was ascribed to the O-H/N-H stretching vibrations (Eskalen, Uruş, Cömertpay, Kurt, & Özgan, 2020). The peak observed around 2922 cm^−1^ can be attributed to the stretching vibrations of C—H (Pajewska-Szmyt, Buszewski, & Gadzała-Kopciuch, 2020). The characteristic absorption peaks at 1680, 1606, 1408, 1290 and 1095 cm^−1^ were associated with the stretching vibration of C=O, C=C, C–N–C, C–OH and C–O–C, respectively and the methylene symmetric bending vibrations was observed at 684 cm^−1^ (Atchudan, et al., 2021).

Additionally, XPS analysis was employed to characterize the chemical structures of BP-CDs. The XPS spectrum (Fig. 2e) reveals that BP-CDs primarily comprise of carbon (C1s, 70.73 %), nitrogen (N1s, 3.85 %), oxygen (O1s, 22.31 %), and sulfur (S2_P_, 2.22 %,). The high-resolution C1s spectrum in Fig. 2f demonstrates three peaks at 284.7, 286.6 and 288.0 eV, representing the forms of C–C/C=C, C—O and C=O, respectively (Ding et al., 2018). The high-resolution N1s spectrum (Fig. 2g) can be fitted one peak at 400.0 eV, indicating nitrogen exists predominantly in the form of pyrrolic nitrogen (Xu et al., 2016). The high-resolution O1s spectrum (Fig. 2h) reveal two peaks at 531.2 and 532.6 eV, which are attributed to C=O and C–O/O–H, respectively. As for the spectrum of S2p, the peaks located at 168.6 eV, 165.9 eV, 163.3 eV and 169.8 eV, 167.1  eV, 164.4  eV are assigned to S2p_3/2_ and S2p_1/2_ as shown in Fig. 2i. The FTIR spectrum and XPS results indicated that BP-CDs contained hydrophilic functional groups like hydroxyl and carboxyl on the surface, which provided their excellent aqueous solubility.

### 3.2 Antioxidant activity of BP-CDs

**Fig. 3:**
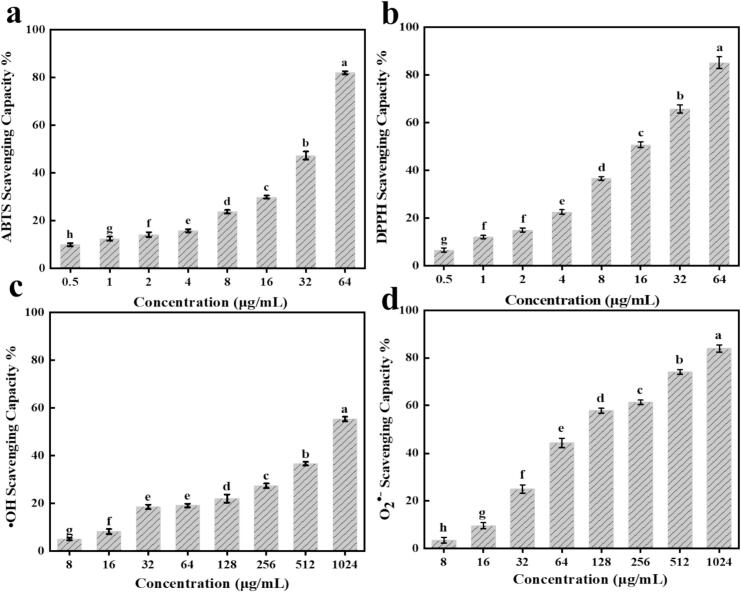
(a) ABTS, (b) DPPH, (c)·OH and (d) O_2_^•−^ radical scavenging activity of BP-CDs. Results are described as mean ± SD of three determinations. Different letters indicate significant differences (*P* < 0.05).

The radical scavenging ability of BP-CDs was determined using different free radicals (ABTS, DPPH, ·OH and O_2_^•−^), which is widely used for evaluating antioxidant activity. As shown in Fig. 3a, a gradually enhancement in the ABTS radical scavenging ability of BP-CDs was found as their concentration increased from 0.5 to 64 μg/mL, showing that BP-CDs scavenge the ABTS radical in a dose-dependent manner. At the concentration of 64 μg/mL, the scavenging capacity of BP-CDs on ABTS reached 81.95 %. A similar result was obtained from the DPPH radical scavenging activity test. BP-CDs scavenge the DPPH radical in a dose-dependent manner at a concentration of 0.5 to 64 μg/mL as shown in Fig. 3b, and at the concentration of 64 μg/mL, the scavenging ability of BP-CDs on DPPH reached 85.10 %. Likewise, it has been demonstrated earlier that the CDs synthesized from coconut shell had great DPPH radical scavenging ability (Chunduri et al., 2016).

It is well-established that reactive oxygen species (ROS) like ·OH and O_2_^•−^ play a significant role in food spoilage (Zhao, Zhang, Mujumdar, & Wang, 2023). In this study, we used Fenton's reaction to generate ·OH and the scavenging effect of BP-CDs on ·OH is presented in Fig. 3d. The ·OH scavenging activity of BP-CDs is in a concentration dependent manner and at a concentration of 1024 μg/mL, 55.34 % of ·OH scavenging was observed. Similarly, the O_2_^•−^ scavenging activity of BP-CDs gradually increases with increase in BP-CDs concentrations (from 16 to 1024 μg/mL), and the O_2_^•−^ scavenging activity was 83.90 % at 1024 μg/mL. Li, et al. also documented that the CDs prepared from *salvia miltiorrhiza* can effectively scavenge free radicals including O_2_^•−^ and ·OH (Li et al., 2020).

The findings revealed that BP-CDs was found to be effective at scavenging the free radicals, suggesting that it possesses strong antioxidant capacity. Carbon nanodots possess surface functional groups such as hydroxyl, amine, and carboxylic acids. In this work, the CDs were observed to be rich in hydroxyl active groups, which can act as proton donors and are capable of transferring free electrons to the CDs bulk structure. In turn, CDs could be degraded by the release of acidic residues such as carbonates and bicarbonates. In this way, CDs are able to scavenge free radicals in vitro (Das et al., 2014). It has been previously shown that garlic and black soya beans were used as precursors to prepare CDs and their antioxidant properties were evaluated. The results revealed both of the CDs possess efficient scavenging activity on free radicals due to the presence of surface groups like carboxyl, amino and hydroxyl (Jia et al., 2019).

### 3.3 Antibacterial activity of BP-CDs

**Fig. 4:**
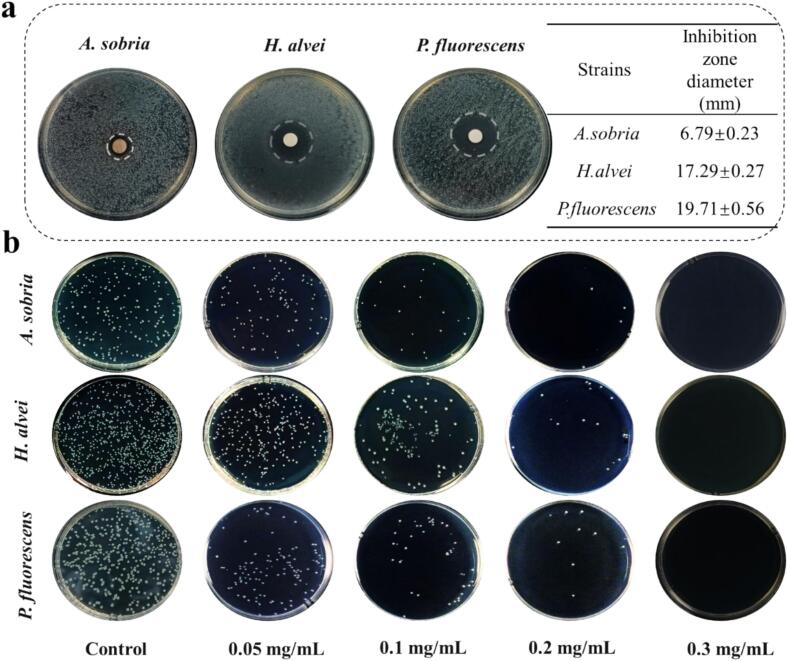
(a) Inhibition zone photos of BP-CDs against *A. sobria*, *H. alvei* and *P. fluorescens* and their quantitative data, and (b) Typical photographs of *A. sobria*, *H. alvei* and *P. fluorescens* after treatment with different concentrations of BP-CDs for 24 h.

To assess the antibacterial activity of BP-CDs, *A. sobria*, *H. alvei* and *P. fluorescens* were selected as tested microorganisms in this work. As illustrated in Fig. 4a, a clear bacterial growth inhibition zone around paper disks for three tested strains were observed, indicating that BP-CDs had antibacterial effects on *A. sobria*, *H. alvei* and *P. fluorescens*. In particular, a better antibacterial activity was observed against *H. alvei* and *P. fluorescens* than *A. sobria*. Specifically, quantitative analysis of the bacteriostatic circles showed that inhibition zone diameters of *A. sobria*, *H. alvei* and *P. fluorescens* were 6.79, 17.29 and 19.71 mm, respectively. In addition, the survival of bacteria treated with BP-CDs for 24 h using the more precise spread plate method. As shown in Fig. 4b, an obviously decreased colonies of bacteria are observed with the increasing concentration of BP-CDs from the LB agar plates experiments. For the three tested bacteria, there were no colonies in the LB agar plates when the concentration of BP-CDs rises to 0.3 mg/mL.

**Fig. 5:**
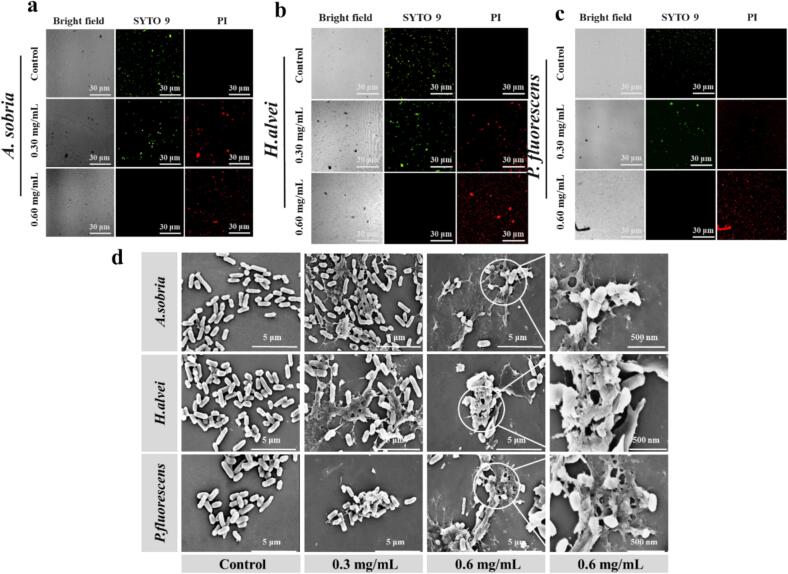
(a, b and c) Fluorescent images and (d) SEM images of *A. sobria*, *H. alvei* and *P. fluorescens* before and after treatment with BP-CDs.

Staining dye PI targeting nucleic acids can penetrate the cell but exclude living cells with intact plasma membranes (Gomes et al., 2018). PI (red) was used to indicate dead bacteria, and SYTO9 (green) was used mark live bacteria. Treatment with the BP-CDs at a concentration of 0.6 mg/mL revealed clear red fluorescence in the broad range of *A. sobria*, *H. alvei* and *P. fluorescens* cells (Fig. 5a, b and c), indicating a significant compromise in the cell membrane integrity compared to the untreated bacteria. Moreover, the structural integrity of the bacterial cells, both untreated and treated with BP-CDs, was examined using scanning electron microscopy (SEM). As presented in Fig. 5d, the control bacteria displayed that the integrity of the bacterial cell membrane of *A. sobria*, *H. alvei* and *P. fluorescens* can be clearly observed. After treatment with BP-CDs, the integrated cell membranes of the three strains were severely damaged and cells showed disruption, collapse, perforation, resulting in the leakage of cytoplasmic components to their surface, which is similar to the effects induced by cationic antibacterial agents (Lee et al., 2020). Wang et al. (2018) found that a kind of CDs derived from *Artemisia argyi* leaves exhibits antibacterial ability against gram-negative bacteria including *Escherichia coli*, *Pseudomonas aeruginosa* and *Proteusbacillus vulgaris* by damaging bacterial cell membranes (Wang et al., 2020). It has been reported earlier that because of their specific physicochemical properties including ultra-small size and abundant surface active groups, CDs can stick to the bacterial membrane through electrostatic interaction with the phospholipids in the membrane of cells, which causes cell membrane rupturing, resulting in bacterial death (Shahshahanipour, Rezaei, Ensafi, & Etemadifar, 2019). In addition to the destruction of the membrane, the action of CDs against bacteria may be related to several other mechanisms, including DNA fragmentation/condensation, the production of ROS and inhibition of bacterial metabolism (Liang et al., 2021; Sun et al., 2021). Our work preliminarily revealed an antibacterial mechanism wherein CDs can bind to and rupture the bacterial cell membrane and cause bacterial death.

Meanwhile, the permeability of the cell membrane serves as an indicator of bacterial membrane integrity, which can be evaluated by the cell membrane relative conductivity. It has been established that a higher relative conductivity indicates severe damage to cellular membranes (Jiang, Wang, Xiang, & Zhang, 2022). As presented in Fig. S1a, the relative conductivity of *A. sobria*, *H. alvei* and *P. fluorescens* dramatically increased as a function of BP-CDs concentration (*P* < 0.05). The result confirm that BP-CDs damaged the cell membrane of the tested bacteria and increased their cell membrane permeability. Additionally, we further examined the bacterial cell membrane integrity by detecting the leakage of intracellular compounds of the supernatant of tested bacteria. The nucleic acid leakage (OD_260_) of bacterial cells caused by BP-CDs was shown in Fig. S1b. After treatment with BP-CDs, the absorbance value increased significantly compared to those untreated bacteria (*P* < 0.05), demonstrating that the cell membrane integrity of the tested bacteria treated with BP-CDs was destroyed, resulting in the leakage of intracellular nucleic acids. Moreover, the protein leakage of bacterial cells caused by BP-CDs was shown in Fig. S1c. The absorbance value at 280 nm exhibited a significant increase concomitant with the rising concentration of BP-CDs (*P* < 0.05), indicating that the cell membrane of the bacterial cells had been destroyed. The results indicated that the integrity of the cell membrane was severely damaged after treated with BP-CDs, causing a large amount of intracellular nucleic acid and protein leakage.

### 3.4 Biosafety evaluation

The cytotoxicity effect of BP-CDs on the 3 T3-L1 was performed to ensure the biological safety in the preservation of aquatic products applications. In Fig. S2, BP-CDs do not elicit much cytotoxicity toward 3 T3-L1 with cell viability of more than 90 % at a concentration of 1.2 mg/mL. At higher concentrations up to 3 mg/mL, the cell viability was still 86 %. These results demonstrate that BP-CDs might be a promising antimicrobial agent with negligible toxicity.

### 3.5 Metabolomics analysis

**Fig. 6:**
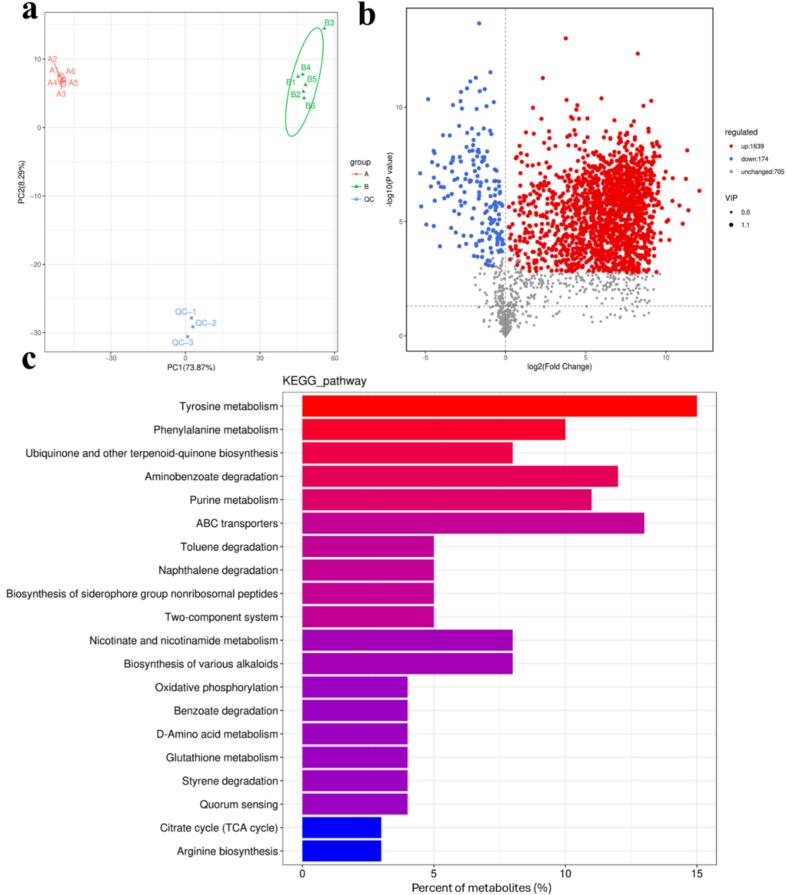
(a) Principal-component analysis (PCA) of metabolite changes caused by BP-CDs treatment, (b) volcano plot for the differential metabolites and (c) the KEGG pathways enrichment analysis between A and B. A: *P. fluorescens*, B: *P. fluorescens* treated with BP-CDs.

To further elucidate the antibacterial mechanism of BP-CDs, we chose untreated *P. fluorescens* and *P. fluorescens* treated with BP-CDs to conduct metabolomic analysis since *Pseudomonas* is a major contributor to the spoilage of aquatic products, meats, and cold ready-to-eat foods. First, principal component analysis (PCA) was conducted to elucidate the alterations in metabolite profiles in response to BP-CDs treatment in *P. fluorescens*. PCA plot revealed all QC samples were clustered together near the coordinate origin, which demonstrated the excellent repeatability of the analysis (Yang et al., 2022). The PCA plot also showed samples from the same group clustered together and good separation between the two groups (Fig. 6a), indicating an obvious difference in metabolite composition between A group and B group. A total of 1813 metabolites differed significantly between untreated *P. fluorescens* and *P. fluorescens* treated with BP-CDs, as shown in a volcano plot in Fig. 6b, among which 1639 metabolites were upregulated and 174 were downregulated. These results suggested that BP-CDs treatment could significantly affect metabolite profiles of *P. fluorescens*. We next discriminated those metabolites by mapping them to KEGG pathways using the pathway enrichment tool in MetaboAnalyst. The results revealed that enriched pathways were related to metabolic processes including energy metabolism (oxidative phosphorylation and citrate cycle (TCA cycle)), substance metabolism (tyrosine metabolism, phenylalanine metabolism, nicotinate and nicotinamide metabolism, purine metabolism and glutathione metabolism), substance transport (ABC transporters) and signal transduction (two-component system and quorum sensing) (Fig. 6c). We further categorized these metabolites based on their database annotation status (KEGG, HMDB, and LIPID MAPS database) and the functional characteristics of their metabolic derivatives. KEGG annotation revealed that many differential metabolites were present in amino acid metabolism, biosynthesis of other secondary metabolite, lipid metabolism, membrane transport, metabolism of cofactors and vitamins and nucleotide metabolism pathways (Fig. S3a), which were essential for bacteria survival. HMDB annotation showed that the metabolites were mainly benzenoids, lipids, and lipid-like molecules, organic acids and derivatives, organic nitrogen compounds, organic oxygen compounds, organoheterocyclic compounds, and phenylpropanoids and polyketides (Fig. S3b). Lipid Maps annotation revealed that the metabolites were mainly fatty acyls, glycerolipids, polyketides, prenol lipids (isoprenoids), sphingolipids and sterol lipids (Fig. S3c). Pathways involved in amino acid metabolism are indispensable for the survival of all living organisms. These metabolic pathways not only furnish energy and intermediate metabolites essential for cellular metabolism, but also plays an essential role in signal transduction, the regulation of gene expression, and antioxidant activity (Zhang et al., 2020). Lipids are important structural components of the cell membrane and maintaining cell membrane integrity is critical for bacterial physiology. The differential metabolites of *P. fluorescens* after BP-CDs treatment are involved in the fatty acid biosynthesis, biosynthesis of unsaturated fatty acids and alpha-linoleic acid, indicating that the bacterial lipid metabolism pathway has been disturbed. ABC transporters are integral to the import of essential nutrients, and notable changes in these transporters could affect the translocation of nutrients and lipids in *P. fluorescens*, as well as the transport of secondary metabolites. Furthermore, pyrimidine and purine metabolism offer structural blocks for nucleic acids synthesis, and a relatively high percent of metabolites involved in nucleotide metabolism in *P. fluorescens* were significantly impacted by BP-CDs treatment, resulting in DNA damage and cell death. In addition, isoprenoids are essential for maintaining bacterial cell physiology, playing crucial roles in the biosynthesis of membranes and peptidoglycan, as well as in electron transport (Abouelhadid et al., 2019). Uridine consumption and uracil production are also linked to the biosynthesis of peptidoglycan, the essential components of the bacterial cell wall (Manzo et al., 2020). These findings indicated that BP-CDs affected the biosynthesis of proteins and lipids and pyrimidine metabolism, destroying the structural integrity of *P. fluorescens* cell wall and membrane and then cell death, which were consistent with the aforementioned results.

## 4 Conclusions

In summary, we fabricated BP-CDs by taking banana peel as a precursor. Detailed characterizations demonstrate the successful formation of BP-CDs. The in vitro antioxidant assays have demonstrated that BP-CDs exhibit exceptional antioxidant activities, effectively scavenging ABTS, DPPH, ·OH and O_2_^•−^. Moreover, the membrane permeability of the bacteria was affected by BP-CDs treatment, resulting in the leakage of the cellular contents. Metabolomics analysis further revealed that BP-CDs treatment led to significant metabolic alterations in *P. fluorescens*, including amino acid metabolism, biosynthesis of other secondary metabolites and lipid metabolism, etc., thus interfering with the normal cell metabolism, inhibiting important cellular physiological activities, and cause cell death. The knowledge obtained from in this work may motivate the sustainable and cost-effective synthesis of CDs with antioxidant and antimicrobial properties, and we hope that BP-CDs can be applied in active packaging development for the preservation of aquatic products as an active agent.

## CRediT authorship contribution statement

**Yueyue Meng:** Writing – original draft, Conceptualization. **Huiyu Zhu:** Methodology, Data curation. **Xinyi Li:** Validation, Software. **Shuang Zhao:** Validation, Software. **Kun Ma:** Software. **Tingting Li:** Supervision, Funding acquisition.

## Declaration of competing interest

The authors declare that they have no known competing financial interests or personal relationships that could have appeared to influence the work reported in this paper.

## Data Availability

No data was used for the research described in the article.

## Appendix A Supplementary data

Supplementary material Image 1
