# Supplementary material for: Antimicrobial activity of carbon dots against aquatic spoilage Bacteria synthesized from Banana Peel waste
Source: Food Chem X. 2025 Mar 15;27:102375. doi: 10.1016/j.fochx.2025.102375 (PMC11981805; doi:10.1016/j.fochx.2025.102375)
Supplement: Supplementary file 1 — Supplementary material [file mmc1.docx]

**Figure captions**

**Figure 1** (a) Relative conductivity, (b and c) nucleic acid and protein leakage of *A. sobria*, *H. alvei* and *P. Fluorescens* before and after treatment with BP-CDs. Results are described as mean ± SD of three determinations. Different letters indicate significant differences (*P* < 0.05).

**Figure 2** Cytotoxicity of BP-CDs on 3T3‐L1 preadipocytes.

**Figure 3** Classification of metabolites according to annotation by (a) KEGG, (b) HMDB and (c) LIPID MAPS database.


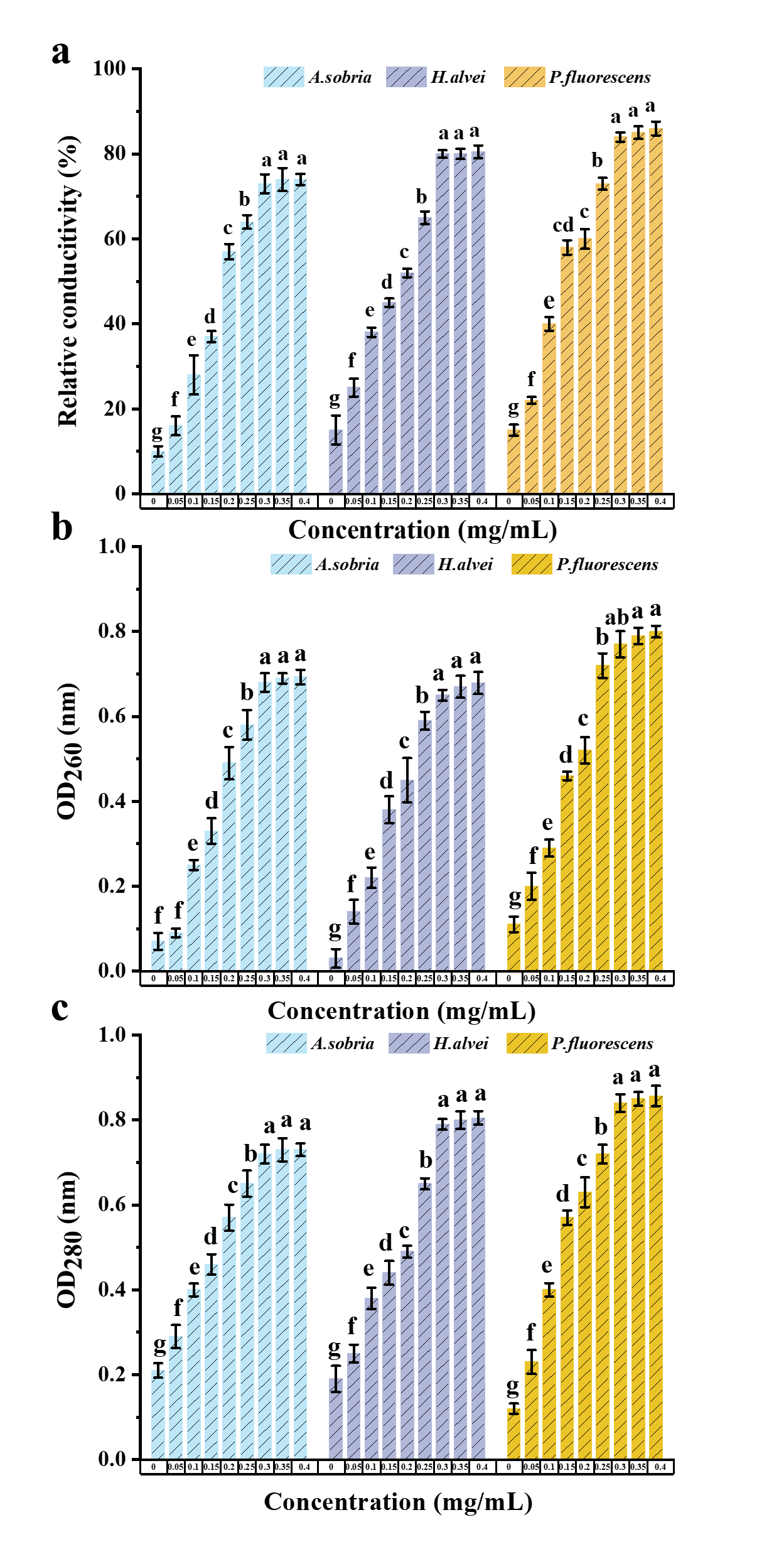


**Figure 1**

**Figure 2**

**
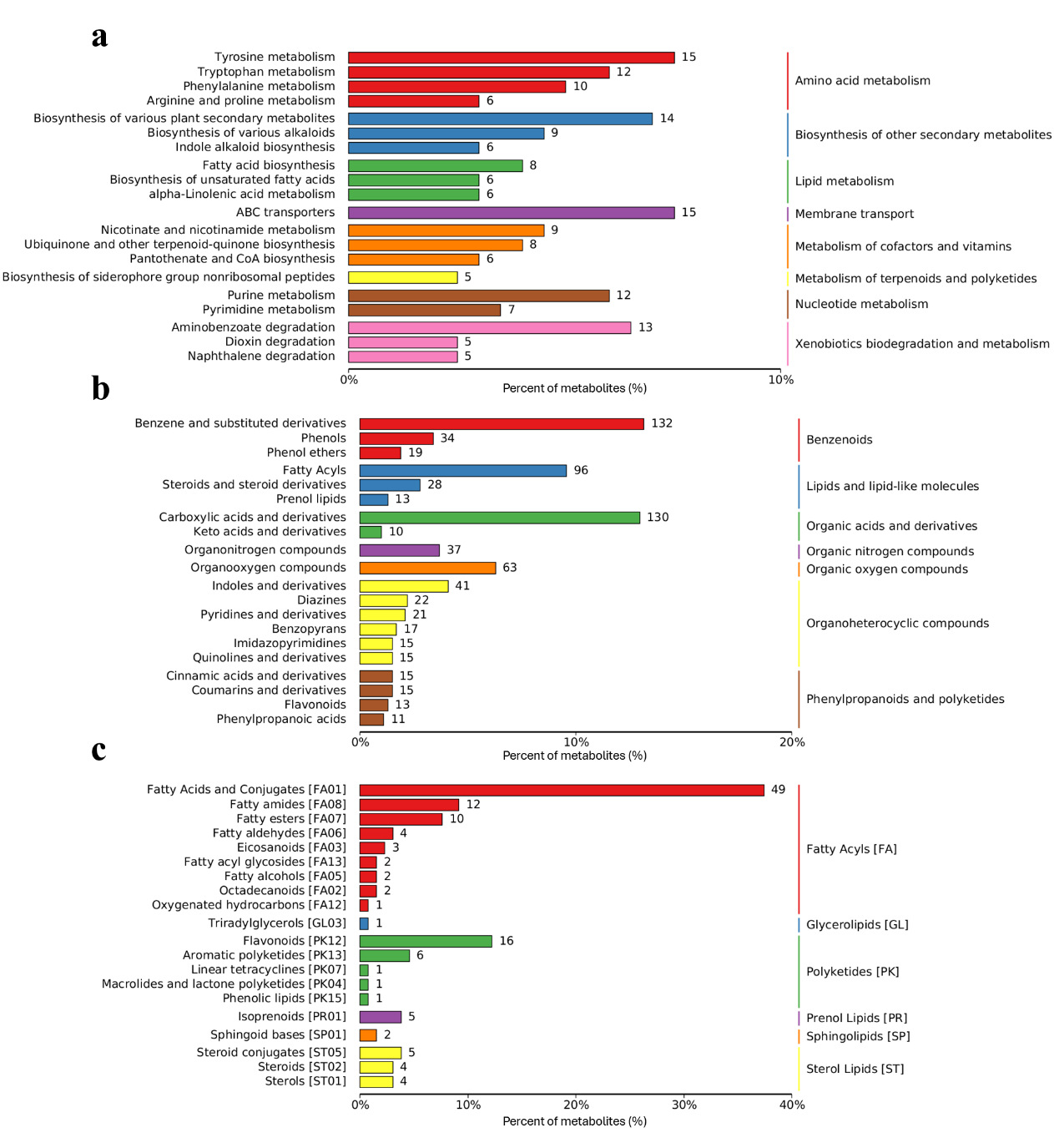
**

**Figure 3**
